# Dementia With Lewy Bodies (Dlb) Diagnosis Rates vs. National Benchmarks: A Completed Audit Cycle in Manchester Memory Services

**DOI:** 10.1192/bjo.2025.10593

**Published:** 2025-06-20

**Authors:** Ahmed Ghaly, Eleni Markopoulou

**Affiliations:** Greater Manchester Mental Health NHS Foundation Trust, Manchester, United Kingdom

## Abstract

**Aims:** Dementia with Lewy Bodies (DLB) is a progressive neurodegenerative disorder accounting for 5–15% of dementia cases. A 2022 audit at Manchester’s Memory Assessment and Treatment Services (MATS) identified DLB diagnosis rates of 2%, significantly below national benchmarks, prompting recommendations for greater awareness and the potential use of the DIAMOND Lewy Toolkit. This re-audit aimed to:

Assess DLB diagnosis rates after initial audit recommendations.

Compare local diagnosis trends with national benchmarks.

**Methods:** A retrospective clinical audit was conducted across MATS Central, North, and South services.

Cycle 1: Data collected for patients diagnosed 1 Nov 2021–31 Jan 2022.

Cycle 2: Data collected 1 Oct 2022–31 Mar 2023.

Inclusion: Patients diagnosed with any dementia subtype within the specified periods.

Exclusion: Mild cognitive impairment, delirium, amnestic disorders, or cases without a definitive diagnosis.

Data source: PARIS electronic patient records.

National benchmarks: Expected DLB rates (5–15%), Alzheimer’s disease (50–75%), Vascular Dementia (≤20%), Mixed Dementia (10%).

**Results:** A total of 493 patients were identified in Cycle 2, with 315 meeting inclusion criteria.

DLB diagnosis rates:

Cycle 1: 2% (n=2/121).

Cycle 2: 2.85% (n=9/315).

Breakdown by team (Cycle 2):

MATS Central: 5% (n=7/133) (↑ from 2%).

MATS North: 1.5% (n=2/129) (↑ from 0%).

MATS South: 0% (↓ from 3%).

Other dementia diagnoses:

Alzheimer’s: 30.7% (lower than expected).

Vascular: 22% (higher than expected).

Mixed Dementia: 28% (higher than expected).

Unspecified Dementia: 16% (higher than expected).

**Conclusion:** While the DIAMOND Lewy Toolkit was available and used, memory nurses apply it only when they suspect LBD, rather than routinely. The increase in DLB diagnosis rates remained modest, and contributing factors may include diagnostic uncertainty, lack of standardised toolkit use, and regional variations in dementia prevalence.

Recommendations:

1. Standardise toolkit use across all clinicians, rather than limiting it to cases where LBD is suspected.

2. Targeted clinician education focusing on recognising early DLB features.

3. Future structured implementation of the DIAMOND Lewy Toolkit and a third audit cycle to evaluate impact.

Further work will explore whether training interventions or mandatory toolkit use could bridge the diagnostic gap and improve DLB recognition.

